# The IFRS adoption, accounting quality, and banking performance: An evaluation of susceptibilities and financial stability in developing economies

**DOI:** 10.1371/journal.pone.0265688

**Published:** 2022-07-29

**Authors:** Chao Ma, Rehmat Ullah Awan, Diandian Ren, Majed Alharthi, Jahanzaib Haider, Robina Kouser

**Affiliations:** 1 School of Economics and Management, University of Science and Technology Beijing, Beijing, China; 2 Department of Economics, University of Sargodha, Sargodha, Pakistan; 3 School of Banking and Finance, University of International Business and Economics, Beijing, China; 4 Assistant Professor in Finance, Finance Department, College of Business, King Abdulaziz University, Rabigh, Saudi Arabia; 5 UniKL Business School, University of Kuala Lumpur, Kuala Lumpur, Malaysia; 6 Department of Economics, University of Sahiwal, Sahiwal, Pakistan; Szechenyi Istvan University: Szechenyi Istvan Egyetem, HUNGARY

## Abstract

International Financial Reporting Standards (IFRS) ’s adoption increased attention to International Accounting Standard Regulations worldwide. It has opened the door for empirical analysis having different perceptions of mandatory IFRS adoption. This paper’s main objective is to examine the impact of accounting quality and IFRS adoption on Pakistan’s banking sector efficiency. We have employed the Malmquist productivity index, Roychowdhury’s Earnings Management, and modified learner index to conduct the empirical analysis. The results mean how much banking sector efficiency is affected by accounting quality and IFRS adoption. The results demonstrate that the banking sector efficiency significantly increases through accounting quality and IFRS. Furthermore, it can be seen that the foreign banks’ efficiency in Pakistan is less than other banks compared to public or private banks. Additionally, more earnings timeliness has been noted in large banks than medium and small banks in Pakistan. Preferably, the practice of quality accounting relies on disclosed information through financial statements. In contrast, the organizations may evade the losses once the information quality is precise and appropriate. The study provides valuable information to managers and other stakeholders.

## 1. Introduction

There is a mutually agreed consensus that the financial crises caused the major obliteration of economic undertakings, histrionic unemployment, poverty, and inequality. These unprecedented unemployment levels, welfare losses controlled by decreasing growth, and strained government investments [1]. Therefore, high quality and worldwide well-accepted accounting standards are required to sustenance the rising market’s globalization [2]. Adopting the International Financial Reporting Standards (IFRS) and accounting quality has also greatly affected its efficiency. Over the past three decades, the banking sector’s efficiency assessment has expanded significantly, and new research and contributions have been added to this relevant knowledge area [3]. It raises serious concerns about the output level attained using the given vector set of inputs, which is a significant problem today [4]. In the past few decades, the issue of measuring efficiency and addressing the differences in productivity between firms has been highly valued by economists. [5, 6] and other authors point to the productivity of a company’s precise core context, for example, management aptitude, the skilled labour and wealth investment, computer technology and R&D investment, invention and corporations structural decisions [7, 8].

Productivity has external variables, for example, technological change, competition, spillovers, flexible market entry, and deregulation [9, 10]. However, there is little evidence of how variations in financial reporting standards and accounting standards impacted its efficiency. Productivity involves the productivity also advantages of the company. Therefore, there is an urgent requirement to recognize how the effectiveness of the business and financial industry is affected by the adoption of IFRS and the quality of accounting [11, 12].

From the perspective of business efficiency, managers’ efficiency and the extensive use of financial statements and disclosed information are described. The literature has hardly mentioned the impact of transition. From an economic perspective, a company’s technical efficiency (TE) is defined as producing a given output level using a given input level or minimal input [13]. The TE concept reflects producers’ ability to waste resources by avoiding the constraints faced by all companies in the group. The first theoretical discussion related to TE measurements exists in [14] thinking. He defines a simple measure of productivity to account for multiple inputs. The authors decompose production efficiency into technical and distribution efficiency in this approach. TE is defined as an enterprise’s ability to maximize output from a given set of inputs, and allocation efficiency is defined as its ability to use these inputs in an optimal ratio that presents their respective prices [15, 16].

However, the above efficiency metrics proposed [17] have been challenged by many researchers who have called for alternatives to improve efficiency. One such alternative is an efficiency measurement based on a directional distance function. The efficiency method based on the direct distance function considers simultaneous improvements in input and output by combining Farrell’s input and output efficiency metrics into one metric. It is necessary to recognize that even the banks considered in this study are homogeneous and are widely protected by the banking industry. However, due to the industry’s heterogeneity, the production technologies they use may not all be unique and may vary in different ownership structures [18].

Conversely, a group boundary (also known as a local boundary) may be formed, belonging only to banks with a specific ownership structure. A meta-boundary or a large border that merges all the banks will be created in addition to this. Therefore, we can evaluate a particular bank for these two boundaries, i.e., group boundaries or local boundaries relative to the meta or boundary [19, 20].

Productivity represents an enterprise’s output achievement measured by consuming the total revenue after a given input (e.g., materials, labour, technology, and other expenses). Simultaneously, efficiency is achieved by destroying the most negligible output to achieve the optimal output level. Financial information is considered to assess the company’s efficiency [21, 22]. However, little work is carried out while examining the efficiency of the heterogeneous accounting system. [15, 23] demonstrated that earnings quality affects manager productivity, and [24] discussed the possibility of companies adopting IFRS after the IFRS is enforced.

There is little evidence of the impact of IFRS on the company’s productivity and its relative position. The economic theory of productivity describes the relationship between inputs and output use. It can evaluate how many lower inputs are needed to produce the desired output level by entering dimensions. You can use the output lens to understand the efficiency of standard input levels and how much output can be produced. [25] argued measurement of efficiency through the various amount of input and output. Accounting statements with different metrics have different reports on managers’ efficiency. For example, on the other hand, the related incentives for performance decline led to inevitable companies. The output and production efficiency have declined.

Conversely, [26] argued that productivity increases when companies are forced to transition after the indigenous Generally Accepted Accounting Principles (GAAP) system adopts IFRS. The GAAP is usually referred to as the United States GAAP system. The primary difference between IFRS and GAPP is that the IFRS is a principal basis while GAPP is rule-based. Under the GAPP system, comprehensive details about transactions are provided, while the IFRS system provides much fewer overall transaction details. Moreover, different interpretations of similar transactions may further lead to extensive disclosures in the financial statement under the IFRS system. So it can be argued that when companies follow the IFRS system, they portray a much more informative and robust balance sheet by permitting companies to show the fair market value of assets less accumulated depreciation.

In contrast, the GAAP system only permits cost information less accumulated depreciation. Therefore, this study’s primary objective is to investigate the banking industry’s performance under the IFRS system. Because of the banking industry’s significance at the international level, various studies focus on more efficient and productive research banks, especially for transition economies and less developed countries. Therefore, the Pakistani banking industry must research from the perspective of IFRS and its efficiency.

The current study has been divided into different sections as the section contains a literature review, section 3 methodology, section 4 contains the results and discussions, and chapter 5 concludes the study.

## 2. Literature review

Various studies are related to our current study’s perspectives on accounting, finance, and new financial efficiency directions [27, 28]. [29] Defined efficiency as an estimate of empirical and optimal values based on the output produced based on a given input level. [30] also argues that the bank efficiency idea must be clearly understood in a realistic model that analyzes bank risk factors. The study regarding bank efficiency [36] emphasized the significance of non-performing mortgages after assessing bank productivity.

In 2007, the global financial crisis (GFC) was primarily caused by a lack of government and central bank oversight, but the poor regulatory environment also "corrupted" the integrity of banks’ internal controls and audits [31]. As a result, a long-term system of management was required. Management control systems (MCS) and the intellectual capital accounting approach in logistics were used for this purpose, and these concepts were tied to the long-term sustainability of company performance. There are many different types of firm resources, including human, physical, and financial ones, evaluated by MCS systems. In quality management, the use of an MCS helps clarify what adjustments are required to maintain high-quality levels [32]. Moreover, the Hungarian self-government system, its fiscal regulations, and the system of obligations and funding have all been aligned following major reforms in 2011 and 2012 [33]. Before overcoming expenses stickiness and enhancing corporate sustainability, manufacturing businesses must implement strategic cost management [34]. [35] computed by applying the Global Reporting Initiative sustainability framework and incorporating information from annual and sustainability reports, such as those on the company’s financial health, social and environmental performance, and the value of specific disclosures (all weighted qualitatively). According to the results of this study, there is a strong correlation between the quality of financial disclosure in the present and the future. As a result, it has a wide range of effects on the economy and society as a whole [36], especially when applied to the retail sector. Accounting and analytical support for agricultural enterprises’ environmental operations [37] and even manufacturing and service organisations’ environmental costs are anticipated to make various charges possible. A company’s ability to manage its impact on the environment is a critical component of its overall success [38].

An associated study [39] and the researchers cited these documents because of Turkey and Malaysia’s economic, social, and political structural affinities and potential similarities (Owusu-Antwi et al. Other studies in Turkey use DEA to evaluate the efficiency of banks. Utilizing the Malmquist DEA model, a value-added approach investigated many Turkish banks’ efficiency and productivity changes. The literature also examines bank efficiency among various countries [40]. For instance, [41] surveyed the Mozambican bank to investigate the banking sector’s comparative efficiency and analyze productivity changes in the banking sector. The outcomes deliver a robust understanding of the banking industry’s scale and efficiency to provide a viewpoint at developing the configuration and economic environment in which banks operate today. In the background of compulsory implementation of International Financial Reporting Standards (IFRS), they scrutinize the association between efficiency and accounting standards’ quality.

Since 2005, many countries have mandated the adoption of IFRS. Previous research [42] has shown that companies required to implement IFRS show equity valuations, more outstanding accounting quality, more considerable market liquidity, and lower equity costs. The researcher predicts the lower error and the dispersion. These gains also depend on a more significant amount on the organization’s human power enforcement in each country [43]. Studied the benefits of adopting IFRS for insurance companies’ performance and concluded that there are three construction methods for using the standard, namely the probability-weighted estimate of future cash flows, discounted. Interest margin risk modification and enduring margin for uncertainty. Often, future profits may cover the cost of adopting the standard by the banking sector [44, 45].

In many developing countries, the quality of local governance institutions is low and, therefore, an essential determinant of adopting IFRS [46]. These countries may suffer from corruption, slow or inefficient government boycotts, or the inability to change. Previous studies regarding the adoption of IFRS partially measure how improvements in the quality of accounting standards affect corporate efficiency. Efficiency metrics are essential for factors that indicate the state of the business.

Most studies are conducted using DEA [47]. In a China Commercial Bank study during 2003–2013, greater efficiency caused greater credit and insolvency risks while decreasing liquidity and capital risk. This research line [48] concluded that commercial banks bear greater credit, bankruptcy, and liquidity risks. Lastly, decreasing liquidity risk increases commercial banks’ market power in the Chinese market (reducing competition). They are convinced that their research offers a widespread understanding of the Chinese banking sector’s risk profile [49]. Studies have examined the effect of risks on banks. However, most of them are concentrated in European banking. Few studies have investigated the impact of trouble on the efficiency of the Chinese banking industry.

We control a series of projections, company and industry-level variables identified in the literature as determinants of management forecast releases based on past research. We include the scale accrual system of accruals to control potential revenue management and the company’s opacity in mandatory financial reporting [50]. When there is an opportunity, the company can provide more voluntary disclosures. Their mandatory reports are of higher quality and control investor demand for more voluntary disclosures [51] measures whether a company is audited by Big Four Auditors, which controls the quality of auditors. Companies with higher auditors may have higher quality financial information and are more straightforward in a voluntary disclosure. Companies in the growth stage have more significant uncertainty and higher information asymmetry, so investors may have higher voluntary disclosure requirements. We also include capitalization and the number of business units the company reports to control information uncertainty and demand. It is speculated that investors facing more significant information uncertainty need more voluntary disclosures, such as management’s forecast of future earnings [52].

The literature lacks the transition impacts of accounting quality, IFRS adoption, and the financial institutions’ productivity from the firms’ efficiency perspective containing the management’s effectiveness. They ignored the comprehensive set of variables to measure the effects of the simulation of efficiency performance. Few studies have inadequate evidence of the efficiencies of financial institutions and their comparative performance.

Our study contributes in the following ways to fill the gap mentioned above. As per the best of the authors’ knowledge, this is the first study providing realistic quantitative confirmation containing the relationship among trilemma assessment of performance, IFRS adoption, and accounting quality by making Pakistan a case study. We have used the Malmquist productivity index, Lerner index indicator of market power, and Roychowdhury’s Earnings Management Model. Unlike others, we have measured Pakistani financial institutions’ efficiency using IFRS adoption and accounting quality effects. Our study is motivated by the current miscellaneous approach to adopting IFRS [53].

Furthermore, associated concerns are considering cross variables studies of (Lin et al., 2019) proceedings [54], facing the issue of inadequate sample sizes that may not characterize a country’s assessment of performance, IFRS adoption, and accounting quality. Our findings focused on the significance of IFRS adoption in Pakistan, making its financial institutions more intensive for investors. Given the benefits allied with Pakistan’s data, the outcomes enhancement and strengthen the results drawn by earlier research using the IFRS adoption and assessment of efficiency.

## 3. Methodology

This study has taken the banking sector of Pakistani as a case study. At the same time, the results show that the IFRS adoption has been considered an independent variable, whereas it has been involved as a dummy factor in the current study. The study used time series data from 2000 to 2019 To conduct the empirical analysis. The study has further distributed data concerning BASEL I (2000–20007), BASEL II (2008 to 2013), and BASEL III (2014 to 2019) to apply quantile regression to specify the role of IFRS on the performance of the Banking sector under those three supervision standards. Furthermore, the efficiency score is calculated to construct a dependent variable, considered the dependent variable. More recently, panel data have been used to examine banking efficiency through the use of various frontier models. There are two primary strategies: linear programming (LP) and parametric frontiers. The stochastic frontier technique [55–57] and data envelopment analysis [58, 59] are frequently used to quantify economic operator performance in a wide range of scenarios. Data envelopment analysis (DEA) is the most utilized LP approach, but free disposal hull (FDH) is rarely employed. Measuring how far an economic agent is from the cost or production frontier is fundamental to efficiency studies. The stochastic frontier approach (SFA) implies that most banks are either on or extremely close to the efficiency frontier (EfF). In a constructed error model, inefficiency—as opposed to normal error—is considered to have a half-normal distribution, allowing for its separation from normal error.

The usage and application of DEA in empirical studies are more accurate; therefore, we considered it more applicable over SFA due to its capability of dealing with numerous outputs and inputs in different measurement units. Also, the SFA does not need any extra information to measure the practical consideration of the frontier. The application of DEA towards the measurement of efficient frontier ensures robust outcomes; thus, we employed the Malmquist DEA productivity method to measure the efficiency of the banking sector in the country.

### 3.1 Efficiency index

Efficiency Index (EI) has been measured through the Malmquist productivity index (EI). EI is a very efficient technique to measure the year-by-year fluctuation in the status of Decision-Making Units (DMU) [60]. The current study investigates and evaluates the efficiency used ‘n’ number of inputs and ‘m’ output. Allocative and technical efficiency has been examined to assess the efficiency situation in the banking sector of Pakistan. Technical efficiency measures the capacity of DMU, which demonstrates that the level of output can be enlarged through the same and assumed input-output, whereas the “comparative *y* efficiency” of a *y*DMU measures the “*y*marginal *y*product” through associating through the situation of “minimum cost”. Malmquist DEA model index is as given

$$M_{t+1}^{icrs}({yy}_{t+1},{yx}_{t+1},{yy}_{t},x_{t})=\frac{yD_{t+1}^{icrs}(yy_{t+1},{yx}_{t+1})}{{yD}_{t}^{icrs}({yy}_{t},x_{t})}\times \sqrt{\frac{D_{t}^{icrs}(y_{t+1},x_{t+1})}{D_{t+1}^{icrs}(y_{t+1},x_{t+1})}\times \frac{D_{t}^{icrs}(y_{t},x_{t})}{D_{t+1}^{icrs}(y_{t},x_{t})}}$$ (1)

Through the Malmquist DEA model, the exterior portion of the brackets are defining the variation in the “technical efficiency”; nevertheless, the equal balanced of an average of two proportions intended into the brackets computes the movement of data between the two time periods ‘stand t + 1’ while the t shows the current year and the ‘t + 1’ shows next year. The progress can be considered in the change of technology. In the given model, the first portion of the right-hand side characterizes a relative change (t+1) regarding the earlier year (t), whereas the ratio’s geometric mean reveals the technological change, which generally shows an innovation effect in the production process DMU. The index constructed through the Malmquist DEA model can decompose into technical change and the relative change in the efficiency change [61].


$$Efficiency\;change=\frac{{yD}_{t+1}^{icrs}({yy}_{t+1},{yx}_{t+1})}{{yD}_{t}^{icrs}({yy}_{t},{yx}_{t})}$$ (2)

$$TECHchange=\sqrt{\frac{{yD}_{t}^{icrs}({yy}_{t+1},x_{t+1})}{{yD}_{t+1}^{icrs}(yy_{t+1},x_{t+1})}\times \frac{{yD}_{t}^{icrs}({yy}_{t},{yx}_{t})}{{yD}_{t+1}^{icrs}(yy_{t},{yx}_{t})}}$$ (3)

In the given Malmquist DEA model, the technical efficiency portion shows xt is the set of vector input during the period of “t”, yt shows the vector set of output during the period of “t.”

In the given direction of time t, “Dt shows the distance function in the objective function during the time of “t”, while “Dt+_1_ = shows the distance function in the objective function during the period of “t+_1_”, “xt+_1_ shows the vector set of input vector during the time previously used to evaluate the efficiency of the banking sector in various countries taking as the case study. Malmquist Data Envelopment Analysis Index is considered a method that helps compute the variation in “Technical efficiencies” (TE) values during two various periods. “Malmquist Data Envelopment Analysis Index” also provides the advantages to compute the relative efficiency of the DMU on production point (t+_1_, X, t+_1_, Y) through the production points (t, X, t, Y). Suppose an improvement has been found in the DMU productivity. In that case, the Malmquist index’s consequence is more extensive than q, whereas if the value of the Malmquist index is less than one, that demonstrates the descending tendency in productivity of DMU furthermore if the Malmquist index outcome is precisely equal to 1 at that moment which translates no changes in efficiency during the period t to t+_1_.

Scale efficiency is an essential tool for measuring efficiency change as defined in the following studies [62]. The effect of IFRS adoption on efficiency has been evaluated by investigating discretionary accruals (expenses) used as a significant proxy of earnings management and accounting quality. The research scholars consider the factor earnings before interest and taxes (EBIT) wherever the outcomes remained accepted. These studies showed that the EBIT impacts discretionary accruals’ values [63]. The modified Jones model has been adjusted to remove the problems noted in the original Jones model, for example, likelihoods towards influence income. The decrease s the given problem through net-revenue, which are considered as accruals “non-discretionary” proxy.


$${NDA}_{t}=\alpha_{1}(1/{Cap}_{t-1})+\alpha_{2}[(\Delta {NetR}_{t}-\Delta {EBIT}_{t})/{Cap}_{t-1}]+\alpha_{3}({PPE}_{t}/{Cap}_{t-1})$$

Δ*NetRt* = variation in net revenue in year *t*

Δ*EBITt* = variation in earnings before tax in time *t* minus time *t*– *1*

*PPEt* = Over-all worth of assets, equipment, and plants at the end of fiscal year *t*

*Capt =* Over-all capital in time *t*

Subsequently, measuring the “non-discretionary” portion of overall accruals, the discretionary portion in a specific period can be calculated by decreasing the NDA (non-discretionary accruals) out of general accruals. Additionally, the overall accruals can be measured through variations in current assets, cash balance, current liability, devaluation, and reimbursement expenses. EBIT consequently, executives and decision-makers can affect EBIT to achieve the desired level of earnings management. Therefore, it can be argued that the control variable deals with the factors such as size, capitalization, EPS, and liquidity risk. These factors can explain the organization’s position. Measurement of the Lerner index is considered the widespread and entrenched factor of market power on individual banks. Learner index imprisonments the power of discrete banks to establish price values overhead marginal cost. If the learner index’s value is higher, there is a more significant bank market power [64]. The fundamental econometric can be used to measure it as follows,

EFFy=∝0y+∝1IFRS+∝2Cap+∝3size+∝4EBIT+∝5DACC+∝6HLI+∝7EPS+∝8LR+ϵ

The given equation EFF shows the efficiency of financial institutions that is major dependent factors, adoption of IFRS shows a global standard of financial reporting, Cap demonstrates a value of capital, EBIT as “earnings before interest and income taxes,” DACC shows the importance of discretionary accruals, HLI can be considered as the hybrid learner index, EPS shows the earning *y*per share, and LR shows the liquidity risk whereas ϵ is the error term. Also, we have used various proxies for specific variables.

### 3.2 Roychowdhury’s earnings management

The word “earnings management” has been employed to explain managers’ decisions to implement the accounting standards during the operational events, directly affecting the earnings to achieve special intentions regarding the financial statements’ results [65]. In sequence, the method of earnings management used can be categorized concerning either effect the procedure of accruals-based accountings. We have used the following techniques can be used to measure earning management,

$$1:\;{abnDiscrExp}_{it}=y\beta_{0}+{y\beta }_{1}{IFRS}_{it}+\beta_{2}{Efficiency}_{it}+\varepsilon_{it}$$

$$2:\;{abnDiscrExp}_{it}={y\beta }_{0}+y\beta_{1}{IFRS}_{it}+\beta_{2}\;{\Delta EBIT}_{it}+\beta_{3}\;(y\Delta EBIT\times {yIFRS\times EPS)}_{it}+\beta_{4}{Efficiency}_{it}+\varepsilon$$

$$3:\;{abnDiscrExp}_{it}=\beta_{0}+\beta_{1}{IFRS}_{it}\times Liquidity\;Risk+\beta_{2}\;{Cap}_{it}\times Learner\;Index+\beta_{3}(Cap\times Size\times {IFRS)}_{it}+\beta_{2}{Efficiency}_{it}+\varepsilon_{it}$$

[66] stated that the extent to which firms deviate from optimal spending through production costs and regression residuals of operating cash flows estimated by industry and annual estimates of current or past earnings. He found that the regression residual is related to the frequency of reaching the income benchmark. Therefore, he believes that the regression residual represents the company’s second-best behaviour in manipulating financial reports. Although later research made some suggestions [67] model is still widely used in the literature.

## 4. Results and discussion

The Empirical analysis is as follows:

Results of the Table 1 show the basic stats of the variables such as the average, maximum and minimum value and standard deviation of discretionary accruals (DACC); hybrid learner index (HLI); earning per share (EPS); liquidity risk (LR) adoption; International financial reporting standards (IFRS); Capitalization; Size and earnings before interest and income taxes (EBIT).

**Table 1:** Summary statistics.

Variables	Average	Maximum	Standard.Dev.	Minimum
DACC	0.085	0.232	0.055	0.001
Hybrid Lerner index	0.073	0.989	0.218	-0.445
EPS	0.174	0.183	1.830	0.080
Liquidity risk	0.092	0.186	0.036	0.034
IFRS	12.18	14.80	1.242	9.098
Capitalization	0.103	0.349	0.064	-0.025
Size	12.18	14.80	1.242	9.098
EBIT	0.028	06.00	0.010	0.004

Table 2 shows the correlation of variables. There was a significant positive correlation between IFRS and size, with a value of 0.393. On the other hand, earnings before interest and taxes are negatively associated with IFRS, having a -0.0708. There is a significant positive correlation between IFRS and freely available accruals, with a value of 0.168. The results show that the earnings per share were negatively associated with IFRS adoption, containing the figure of -0.194. There was a significant positive correlation between liquidity risk and IFRS, with 0.195. The scale efficiency was negatively correlated with liquidity risk, EBIT, and Lerner index, respectively, which were -0.460, -0.578, and -0.198. The accrual basis was negatively associated with the liquidity risk and is shown as -0.00766. Four reservoir poles have been formed in the case of a change efficiency. There is a significant negative correlation between earnings per share and liquidity risk, with a liquidity amount of -0.263. Therefore, it is all related to the association between variables.

**Table 2:** Pearson correlation.

Variables	*y*IFRS	*y*Cap	*y*Size	*y*EBIT	*y*DACC	*y*HLI	*y*EPS	*y*LR
*y*IFRS	1							
*y*Cap	0.0119	1						
*y*Size	0.393***	0.0505	1					
*y*EBIT	-0.0708	0.0208	0.578***	1				
*y*DACC	0.168**	0.133*	0.112	0.00139	1			
*y*HLI	-0.180**	-0.0708	0.460***	0.436***	0.0369	1		
*y*EPS	-0.194**	0.00362	0.0584	0.0567	-0.148*	0.0563	1	
*y*LR	0.195***	0.0475	0.198***	0.132*	0.00766	0.198***	0.263***	1

*LR liquidity risk, HLI hybrid learner index, ***y*** Cap shows the ***y*** capitalization.

Results in Table 3 show that the various efficiencies change in different banks, listed and classified accordingly. This change in efficiency is derived from the Malmquist DEA model investigation of multiple banks. Detailed results show that the bank’s efficiency changes according to the country’s environment. The efficiency change of these banks having a value of less than 1 indicates the banking sector’s inefficient bank. If the efficiency value is 1, struggles are being put to attain higher efficiency.

**Table 3:** Productivity change.

Banks	EC	Tech C	PEFC	SEFC	TFPC
PVT banks	1.019	0.996	1.010	1.009	1.015
SO banks	1.009	1.003	1.012	0.997	1.012
Isl Banks	1.012	1.007	1.010	1.002	1.019
FRN Banks	0.993	0.993	0.994	0.999	0.986

Note: Here, EC = Efficiency Change; Tech C = Technologcial Change; PEFC = Pure Efficiency Change; SEFC = Scale Eficiency Change; TFPC = Totla Factor Productiviey Change.

As concerning the private banks, the total factor productivity of all these banks has changed by more than one. Now turning to Islamic banks, Pakistan’s Islamic banks are limited, with fewer than 1change in overall efficiency. Therefore, it is not efficiently associated with Islamic banks. However, Islamic banks have an average score greater than 1.0 and a score of 1.019. The average overall efficiency changes for foreign banks is less than 1, so they are very inefficient compared to other banks.

Table 4 shows that all banks have profitability and timeliness, which means that the β2 coefficient is positive at 25, 50, and 75 percentiles and is statistically convincing. Through observations and pseudo R^2^ values, it can be seen that the timeliness of large banks is more timely than that of small and medium banks. First, the adoption of IFRS has led to an increase in information transparency and disclosure, which in the sequence has reduced information irregularity and made equity funding easier. Subsequently, it must also increase contracts’ productivity between corporation’s administrators and encourage managers to ensure stockholders’ best interests. Better information also helps the board and other third events measure, reducing chances of managing resourcefulness, such as escaping and wasting resources. Third, the increase in comparative results from the mandatory adoption of IFRS can increase institutional shareholdings through official investors and external pool funds, resulting in more effective relative performance assessments and increased competition in the product market. Our experience has shown that the productivity of mandatory adopters using IFRS is significantly improved. Reports on financial statements have been debated since the 1930s. The growth of the global market has led the primary function of IFRS is to amplify the parallelism of financial statements and improve the transparency of the company and the quality of financial reporting. We recommend that by improving the quality of audits, its targets can be more effectively achieved. Accounting quality can be conditional as an opportunity for auditors to discover material misstatements due to accounting irregularities.

**Table 4:** Quintile regression results.

3Dependent 3Variable: Earnings									
	3Islamic			3Commercial			3Public		
Variables	3q25th	3q50th	3q75th	3q25th	3q50th	q75th	3q25th	3q50th	q75th
IFRS1	330.09	30.117	37.62	35.628	34.289	32.82	311.87	30.034	32.66
	3 (1.95)*****	3 (1.23)*	3 (1.21)*	3 (0.38)*	3 (1.99)*****	3 (1.31)*	3 (1.28)**	3 (0.98)*	3 (1.04)*
IFRS2	3–0.584	31	31.129	33.713	31	31	314.17	37	1.942
	3 (0.64)*	3 (59.82)*	3 (2.47)**	3 (0.79)*	(3.59)*	3 (3.59)*	(4.35)*	3 (2.99)*	3 (1.17)*
IFRS3	20.86	31	31	7.014	4.412	1	-29.91	9	4.45
	3 (1.86)****	3 (2.43)*	3 (0.14)*	3 (0.65)*	3 (2.51)**	3 (2.64)*	(3.27)*	(1.95)****	3 (1.79)*
3Pseudo R^2^	0.111	30.291	30.129	30.016	0.122	30.11	30.099	0.056	0.42
	3**Investment**			3**MFB**			3**Specialized**		
	3**q25th**	3**q50th**	3**q75th**	3**q25th**	3**q50th**	3**q75th**	3**q25th**	**q50th**	3**q75th**
IFRS1	3117.4	377.85	3–64.6	3.861	30.991	30.123	5.747	30.001	0.836
	3 (3.35)*	3 (3.55)*	(2.08)****	3 (6.35)*	(0.62)**	3 (0.32)*	3 (0.08)*	(0.22)*	(0.15)*
IFRS2	319.11	314.41	312.44	30.081	30.17	30.131	2.972	1.079	0.993
	3 (3.28)*	3 (2.81)*	3 (1.90)*****	3 (0.09)**	3 (0.25)*	(0.21)*	3 (5.14)**	3 (2.55)**	(10.77)**
IFRS3	353.26	335.82	330.48	4.352	31.094	0.125	5.935	1.079	31.407
	3 (2.89)*	3 (3.50)*	(2.08)****	(11.26)*	3 (1.01)*	(0.29)*	3 (0.17)*	3 (0.29)*	3 (0.52)*
Pseudo R^2^	30.308	30.247	0.832	0.173	0.013	30.132	0.217	0.168	0.191

3Note

* 3P-value = 0.01

**3 P-value = 0.02

*** 3P-value = 0.03

**** P-value = 0.04, ****3P-value = 0.05.

1. Extension method calculates the error as an incorrect amount for the current income statement. Constant use of the balance method will ultimately lead to the accrual of material miss-statements on the financial information.
2. Iron Curtain Method: The iron curtain method mainly concentrates on financial information when the income statement focuses on the carry-over process. This method will not reproduce the current time balance sheet due to apprehensions about corrections to misstatements in previous years.

### 4.1 Discussion

The transparency of financial reporting is critical to ensuring the proper functioning of financial markets. Recently, the lack of transparency of some of the largest multinational companies has caused the collapse of markets. These consequences include bribery, money laundering, and general corruption, totalling billions of dollars. Besides, they highlight the greater extent and potential impact of impenetrability in emerging markets. Therefore, regulators must focus on policies that are transparent to financial disclosure. Thousands of financial statement data were extracted from the Thomson Reuters Eikon database to calculate accruals and the company’s total assets. The database also includes bid-ask spreads, market capitalization, the audit firm’s identity, whether the financial statements meet IFRS requirements, and listed on the exchange through depositary receipts. Other company characteristics, such as ownership concentration among the largest shareholders. The calculation of proportional accruals is described as an extended Jones model estimated to obtain a measure of income opacity. The IFRS National Profile shows that some companies have been deferred until 2010. In Brazil, since the two-year transition period since 2008, IFRS has become a mandatory requirement for non-financial companies since 2010.

The fact is that the adoption date is different, so its transition period helps determine the significant impact of the benefits. IFRS uses date cross-section and time variation, while the IFRS indicates the remaining variables’ partial impact. The specifications are identical to the equations, which only control the adoption of IFRS and auditing quality. Then use the company’s other features to extend the regression to measure the liquidity by the spread. Further, it can be seen that the expands the equation through macroeconomic controls: the growth of (GDP) and the spread of the emerging market bond market. These estimates confirm our main findings. Part of the impact of the audit quality variable is negative, while the total effect of adopting IFRS is positive. These two estimates are statistically significant and reliable in several competing specifications.

In contrast, some of the other conditions that result from the adoption of IFRS are not statistically significant, which means that once we apply the scope of the new regulations to other company attributes. Expose the impact of adopting new rules. Review quality and larger companies are less opaque than smaller ones, but we find no significant relationship between liquidity and ownership concentration. However, managers who choose one of them to calculate non-current assets may be based on different motives, income increases, or income reductions, which affect the company’s financial situation.

The first step is for companies with publicly listed securities to implement IFRS as soon as possible. The next step is for the trading company to implement IFRS. All publicly-traded companies must go to the next phase. There are two advantages to encouraging trading companies to adopt the IFRS standard before 2005: present more open financial reporting; early adopters’ expertise has helped other publicly traded corporations switch. The multi-agency structure of the accounting environment is another critical issue that must be addressed before IFRS can be implemented. The capital market had the most significant influence on companies who voluntarily implemented IFRS, as they discovered when comparing required and voluntary adopters. The impact of stock market liquidity, equity costs, and stock values was examined using a business panel regression with company fixed effects. After the IFRS report was made required, the market mobility of mandated adopters surged considerably. In addition, they discovered that capital market profits could be generated when a reasonably tight national and institutional context encourages a strong company transparency incentive.

In addition, nations with significant discrepancies between local accounting practices and IFRS and countries that do not implement IFRS convergence initiatives in advance have a greater impact. IFRS supporters believe that mandatory adoption of uniform accounting standards can improve financial statements’ comparability and thus attract more cross-border investment. Compulsion by regulators to adopt International Financial Reporting Standards (IFRS) boosted mutual fund ownership in other countries as well.

## 5. Conclusion

The study’s objective is to check whether the efficiency can be improved with accounting quality and IFRS adoption. The results show that efficiency is enhanced by adopting IFRS and optimizing its audit quality. There is a significant positive correlation between IFRS and discretionary weights, and there is a significant negative correlation between earnings per share and IFRS. There was a significant positive correlation between liquidity risk and IFRS, with 0.195. The scale efficiency was negatively correlated with liquidity risk, EBIT, and Lerner index, which were -0.460, -0.578, and -0.198. The accrual basis was negatively associated with the liquidity risk and is shown as -0.00766. Four reservoir poles have been formed in the case of a change in efficiency. The difference in the average total factor productivity of Islamic banks, private banks, and public banks is that it maintained good performance in the banking sector. While the total factor productivity of foreign banks changes by less than one point, so in comparison, its productivity is very low. Other banking industries.

Our research provides more insight into whether high-quality accounting standards can increase a company’s productivity and efficiency. The survey results show that their production efficiency will increase for companies with high accounting quality and adopting IFRS. These banks are more efficient, and their efficiency changes to 1 or greater than 1. The advantages of IFRS adoption are as follows,

- Use a well-accepted global reporting standard that adds to the practicality of financial reporting.
- Investors will get better decision statistics and knowledge about the firm.
- The company will have greater flexibility in relevant accounting principles.
- Need to reduce the complexity of financial reporting.
- Management at all levels would be involved in financial reporting standards and understand those transactions.
- Finally, businesses must be much more efficient to enjoy the advantage of cost savings.

These advantages can be realized when the company adopts IFRS and implements undertakings following the guidelines. Besides, the findings provide valuable guidelines for decision-makers and determine the extent to which existing GAAPs and IFRSs are adopted or integrated by presenting another measurement of effect calculation.

### 5.1 Limitations and future research directions

The study’s limitation is a small sample size unique to the banking sector in Pakistani. From the perspective of efficiency, this paper discusses the impact of IFRS at the enterprise level. It is the concern of enterprises in expressing their persistence and development policies to meet globalization’s problems. Despite the mandatory adoption of IFRS, our research has made a wide range of contributions in this direction. However, the study delivers supportive indications towards the adoption of IFRS.

Upcoming work can be extended to investigating other businesses and production sectors with different contexts, which need to continue discussing IFRS implementation’s significances from an efficiency perspective. There is still plenty of room for further research investigating the correlation between accounting information and reporting efficiency under different reporting standards. Besides, deposit banks should reinforce their credit risk management and reporting approaches following International Financial Reporting Standards. It will ensure the excellent quality of assets in the stated financial statements. It would remain motivated to break down the impact of efficiency gains into components inside and concerning industries. After the mandatory adoption of IFRS, it is interesting to examine the manager’s investment behaviour and assess their contribution to improving its productivity. The IASB has carried out an improvement project that varies by time to increase efficiency. The IFRS guidelines are being reviewed in this situation to do future research in diverse time frames. Additional research can be completed by increasing the sample size and adding more countries using the adoption of IFRS. It can also remain compared. Therefore, there are all suggestions here in the study.
